# Revisiting the technical validation of tumour biomarker assays: how to open a Pandora's box

**DOI:** 10.1186/1741-7015-9-41

**Published:** 2011-04-19

**Authors:** Caterina Marchiò, Mitch Dowsett, Jorge S Reis-Filho

**Affiliations:** 1 Department of Biomedical Sciences and Human Oncology, University of Turin, Via Santena 7, 10126 Turin, Italy; 2 Academic Department of Biochemistry, The Royal Marsden Hospital, Fulham Road, London, SW3 6JJ, UK; 3 Molecular Pathology Laboratory, The Breakthrough Breast Cancer Research Centre, Institute of Cancer Research, 237 Fulham Road, London, SW3 6JB, UK

## Abstract

A tumour biomarker is a characteristic that is objectively measured and evaluated in tumour samples as an indicator of normal biological processes, pathogenic processes, or pharmacologic responses to a therapeutic intervention. The development of a biomarker contemplates distinct phases, including discovery by hypothesis-generating preclinical or exploratory studies, development and qualification of the assay for the identification of the biomarker in clinical samples, and validation of its clinical significance. Although guidelines for the development and validation of biomarkers are available, their implementation is challenging, owing to the diversity of biomarkers being developed. The term 'validation' undoubtedly has several meanings; however, in the context of biomarker research, a test may be considered valid if it is 'fit for purpose'. In the process of validation of a biomarker assay, a key point is the validation of the methodology. Here we discuss the challenges for the technical validation of immunohistochemical and gene expression assays to detect tumour biomarkers and provide suggestions of pragmatic solutions to address these challenges.

## Introduction

Biomarkers are the defining facet of translational cancer research; however, there is a great deal of confusion about the actual definition of what a biomarker is and what its characteristics are. Arguably, the most widely accepted definition is the one put forward by the Biomarkers Definitions Working Group, which defines a biomarker as "a characteristic that is objectively measured and evaluated as an indicator of normal biological processes, pathogenic processes, or pharmacologic responses to a therapeutic intervention" [1]. As the definition suggests, biomarkers can be used for multiple purposes in cancer research and measured in distinct types of specimens, such as tissue samples as well as peripheral blood (see, for example, circulating tumour cells), by using several assays. Despite the existence of excellent guidelines for the development and validation of biomarkers [2-4], there is a great deal of confusion when it comes to determining the validity of an assay to detect a biomarker. The process of biomarker development is by no means trivial; for the purpose of simplicity, it can be broadly divided into four main phases: (1) discovery of a potential biomarker through hypothesis-generating preclinical or exploratory studies; (2) development and technical validation of the assay for the identification of the biomarker in clinical samples; (3) demonstration of the biomarker's potential clinical utility, first in retrospective analyses and subsequently in prospective studies; and (4) continued assessment of the validity of the biomarker in routine clinical practice (Table 1). The term 'validation' in the context of clinical studies has changed dramatically over the years; currently, perhaps the most adequate definition for a valid biomarker is a biomarker that is fit for purpose [5].

**Table 1:** Overview of the phases of biomarker development and validation^a^

Phase	Means/instruments	Main challenges and sources of bias
Discovery of a potential biomarker	Hypothesis-generating preclinical or exploratory studies	Selection of biomarker based on the availability of antibodies on the market
Development and technical validation of the assay for the identification of the biomarker	Optimisation of IHC-based assays for formalin-fixed, paraffin-embedded samples	- Use of clinical samples not suitable for the analysis (for example core biopsies instead of surgical samples and TMA instead of full sections) - Lack of reliable positive and negative controls - Poor fixation of clinical samples - Wrong antigen retrieval procedure - Wrong detection method Misinterpretation of the results - Training/competency of the staff - Suboptimal performance of the antibody due to poor fixation of archival tissues (in particular for retrospective studies)
Validation of the clinical significance of the biomarker	First retrospective studies and subsequent prospective studies	- Training/competency of the staff - Use of small cohorts or large cohorts that include series of cases in which the biomarker has been previously validated
Continued assessment of the validity of the biomarker in routine practice	Internal and external quality assurance program	- Poor participation/adhesion to the programme - Lack of competency of pathologists participating in the program - No action taken if failing quality assurance

^a ^Description of the phases of biomarker development and validation, and the main challenges and potential sources of bias, using immunohistochemistry-based

assays as a paradigm.

IHC: immunohistochemistry; TMA: tissue microarray.

Although great emphasis is given to the discovery and validation of the clinical significance of the biomarker, the technical validation of assays for novel biomarkers has not been embraced with the same enthusiasm, probably because of its more technical and apparently less rewarding nature. Nonetheless, the process of assay validation is critical for the introduction of a new biomarker in routine clinical practice. This minireview focuses on the technical issues related to validation of biomarkers analysed directly in human tumour tissue samples, with breast cancer pathology serving as a paradigm. It should be noted, however, that the concepts discussed in this review are applicable to biomarkers based on other types of samples (for example, circulating tumour cells, blood, serum, urine and other bodily fluids).

## Validation: when and why?

A biomarker often fails to be incorporated into clinical practice, not because of flawed science underpinning its discovery but because of poor choice of the assay used for its detection and inadequate validation of its accuracy [6,7]. For the successful use of a biomarker assay in clinical practice, it is of paramount importance that the testing of the assay employ robust reagents and be based on a reliable and robust technology. Several false dawns in translational research have stemmed from attempts to introduce a technology that was not sufficiently mature at the time and from the sources of technical bias not being entirely known (for example, mass spectrometry-based serum proteomic analysis for the diagnosis of ovarian cancer) [8,9].

Methodological validation needs to be considered both in well-established and in new assays. In the first scenario, as the assay is already introduced into clinical practice, ongoing demonstration of validity through in-house and external quality assurance schemes allows false-positive and false-negative results to be minimised. The importance of this problem is placed in stark focus by recognising that these checks are necessary to ensure that potentially life-extending or lifesaving therapies are not denied to patients who may benefit from them and that patients are not unnecessarily exposed to toxicities or given false hope. Several initiatives have been introduced to provide guidelines for testing the adequacy of the routine biomarker tests (that is, oestrogen receptor (ER), progesterone receptor (PR) and human epidermal growth factor receptor 2 (HER2) tests) in breast cancer (Table 2). In the United Kingdom, the National External Quality Assessment Service programme organises four assessments every year that include antibody testing on multi-tissue and cell line blocks, an expert review, a confidential report, online images and detailed analysis of data regarding methods [10]. Importantly, these approaches have led to a significant increase in the number of laboratories offering optimal quality immunohistochemical assessment of these markers [10]. External quality assurance does not, however, replace the need for internal quality control measures that enable individual batches of samples to be accepted with confidence or, alternatively, rejected. It should also be noted that internal controls in individual samples, represented by the presence of normal tissue adjacent to the tumour, can be of great support to define the validity of a test. For example, in the assessment of ER, PR and HER2, it is crucial to select a tumour block that, in addition to the tumour areas, also contains adjacent normal ducts or lobules which can be used as internal controls.

**Table 2:** Examples of external quality assurance schemes for routine biomarkers employed in breast cancer pathology^a^

QA scheme	Scope	Website
ASCO/CAP	To improve the accuracy of test results and ensure that patients receive appropriate care	http://www.cap.org/ http://www.cap.org/apps/cap.portal
UK NEQAS	To promote optimal patient care by facilitating the availability of reliable laboratory investigations, through provision of objective information on participant performance and professional advice and assistance where appropriate	http://www.ukneqas.org.uk/
NordiQC	To promote the quality of IHC by arranging schemes for pathology laboratories, assessing tissue stains, giving recommendations for improvement and providing good protocols	http://www.nordiqc.org/
Canadian external quality assurance program	To systematically monitor and improve the proficiency of IHC testing laboratories and those involved with IHC testing nationwide	http://www.ciqc.ca/
The RCPA Quality Assurance Program	To provide external proficiency testing for histopathology laboratories in the areas of diagnostic and technical expertise	http://www.rcpaqap.com.au/

^a ^Names, scope and websites of the main external quality assurance schemes for breast cancer biomarkers based on immunohistochemical and *in situ *hybridisation assays.

QA: quality assurance; ASCO: American Society of Clinical Oncology; CAP: College of American Pathologists; UK NEQAS: United Kingdom

External Quality Assessment Service; IHC: immunohistochemistry; NordiQC: Nordic Immunohistochemical Quality Control; RCPA: Royal College

of Pathologists of Australasia.

The early technical validation of a new biomarker assay is critical to minimise waste of resources, generation of misleading results and possibly disrepute. In this context, however, full validity is often difficult to prove, given that these assays come directly from research assays for which a gold standard is usually not available.

## Validation of assays for novel biomarkers

Immunohistochemistry, fluorescence and chromogenic *in situ *hybridisation, expression profiling, either microarray-based or performed by quantitative real-time reverse transcriptase-polymerase chain reaction (qRT-PCR), and mutation analysis represent the main techniques currently being introduced into everyday practice in pathology laboratories. Among these techniques, immunohistochemical tests remain the most widely used in routine practice and, importantly, in the assessment of biomarkers in translational research endeavours. We therefore focus primarily on their validation.

**Figure 1:**
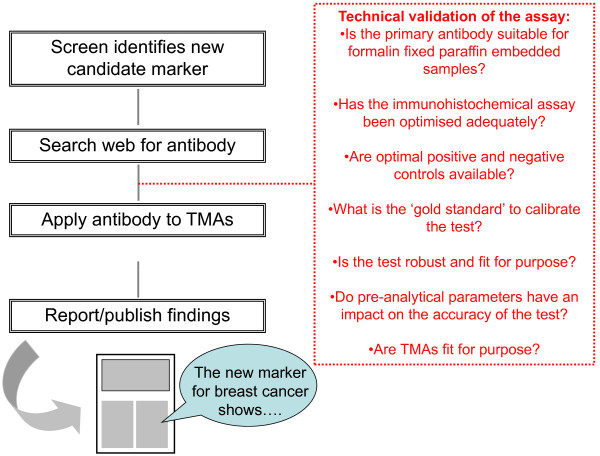
**Schematic representation of the validation process of novel biomarkers by using immunohistochemistry**. A typical scenario for biomarkers evaluated by immunohistochemistry contemplates that new candidate markers are identified through screen analyses. Then, if commercially available antibodies specific to those markers are found, sections of tissue microarray (TMA) blocks containing a large number of samples are used to prove preliminary results. Whenever a new antibody is used to test a novel biomarker on tissue samples, technical validation is mandatory.

In the case of immunohistochemistry, a typical 'quick and easy' approach is illustrated in Figure 1. It should be noted, however, that such an approach carries with it far more pitfalls than many investigators assume. Although immunohistochemistry has been introduced in pathology laboratories for more than 25 years and is a relatively user-friendly technique, one should not forget that the results of immunohistochemical analysis may be affected by a series of preanalytical and analytical parameters [11-15].

Preanalytical variables are the 'weakest link' of immunohistochemistry, as the properties of the tissues analysed and, consequently, the results, may be affected by several factors, including the time to collection (that is, the length of time that tissues are subjected to warm ischaemia between removal of the tissue at surgery and fixation), details of fixation (type of fixative agent used and length and conditions of fixation), dehydration steps, and conditions for paraffin-embedding (temperature of the paraffin). These preanalytical parameters are beyond the control of investigators, are most often unrecorded, and constitute a major potential source of bias, in particular in multicentre, retrospective studies. For example, the time to fixation and its length have been branded as the 'Achilles heel' of phosphoprotein assessment in clinical specimens [16] as recently shown by Pinhel and colleagues [16,17], who found consistently significantly lower levels of the phosphoepitopes in surgical specimens compared with those found in core biopsies.

Four main analytical issues require special attention. Antigen retrieval (that is, a method that enables immunohistochemistry to be applied to formalin-fixed, paraffin-embedded (FFPE) samples), type of detection system, the choice of antibody, and the material to be used [11-13]. Enzyme-based and heat-induced epitope retrieval are available, for which strict protocols should be followed to obtain accurate results [11]. Excellent reviews have described the pitfalls of antigen retrieval and how they can be overcome [11-15]. Suffice it to say that in the absence of optimal positive and negative controls and a 'gold standard', changing antigen retrieval settings can render a given case positive or negative.

**Figure 2:**
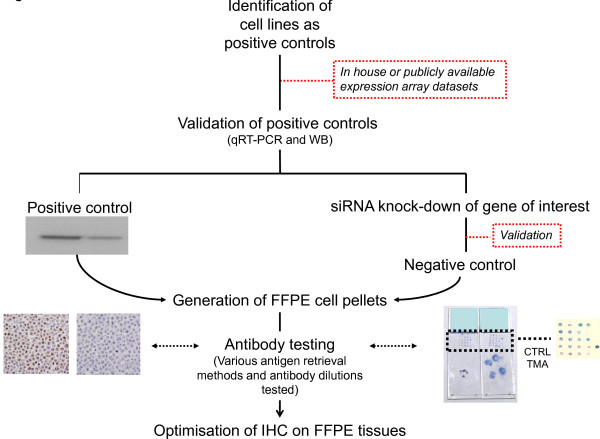
**Schematic representation of a suggested approach to adopt in the optimisation of 'research-only' antibodies**. To generate ideal controls for the optimisation of antibodies for immunohistochemical analysis of formalin-fixed, paraffin-embedded tissues, we propose the use of optimal controls in the form of cell lines. First, cell lines that overexpress the gene and protein of interest are identified by mining publicly available databases (for example, microarrays and proteomics). The expression of the gene and protein in the cell lines identified as 'overexpressors' is validated by Western blot analysis and quantitative real-time reverse transcriptase-polymerase chain reaction (qRT-PCR) assay. These validated cell lines are used as positive controls. Once this validation step is completed, the gene of interest is silenced using validated short interfering RNA (siRNA}. The process of gene silencing is subsequently validated by qRT-PCR assay and Western blot analysis, and these cells are used as negative controls. After *in vitro *validation, pellets of the positive and negative control cell lines are produced and then subjected to formalin fixation and paraffin embedding using routine methods. These controls are then used for optimisation of the antibody titration and choice of antigen retrieval system. Please note that the siRNA negative control has an internal positive control, given that the efficiency of siRNA silencing almost never reaches 100%. In addition, multi-tumour blocks and tissue microarrays constructed with distinct types of tissue can be used as positive and negative controls (images of routine diagnostic slides courtesy of CM). CTRL: control; FFPE: formalin-fixed paraffin-embedded; IHC: immunohistochemistry; siRNA: short interfering RNA; TMA: tissue microarray; WB: Western blot analysis.

Arguably the most challenging aspect of any immunohistochemical assay is the choice of antibody. Before applying 'research-only' antibodies to FFPE tissues, their sensitivity and specificity need to be determined. It should be emphasised that this cannot be achieved merely by evaluating the sensitivity and specificity of the antibody by means of Western blot analysis; these parameters need to be assessed in FFPE samples and using optimal controls (Figure 2). It is always difficult to find the optimal balance between sensitivity and specificity, and, in this respect, the antigen retrieval method and antibody titration are critical, as is the availability of optimal controls to be used as a gold standard. One helpful and informative approach uses in-house or publicly available expression array data sets to identify cell lines that can be exploited as positive controls and then to validate their use as positive controls on the basis of qRT-PCR assay and Western blot analysis. Negative controls can be generated by siRNA (short interfering RNA) knockdown of the gene of interest in the cell line. Subsequently, antibody testing can be performed on FFPE cell pellets, and finally optimisation of immunohistochemistry on FFPE tissues will follow (Figure 2). In this process, the type of detected subcellular localisation may also help the researcher understand whether the antibody is specific. Finally, when dealing with biomarkers used in routine diagnostic practice, audit of the results from annual workload can provide supporting evidence of the actual performance of an optimised assay used in routine practice.

Another crucial aspect is the type of tissue to be used. Tissue microarrays (TMAs) have become very popular in studies aiming to determine the distribution of a given marker in a cohort of samples [18,19]. Although TMAs have proven to be excellent tools, they should be employed for the testing of biomarkers whose expression is relatively homogeneous and should be used only if the concordance between the results of the analysis of a given marker on whole tissue sections and TMAs is close. Regrettably, assessing the latter appears not to be a common practice. It should be noted that this issue is applicable even to well-validated tests in breast cancer research and practice (for example, the discordant results in the analysis of PR expression in TMAs vs. whole tissue sections [20]).

## Gene expression profiling studies: microarrays or micro-awry?

In recent years, microarray gene expression profiling and its derivatives have been widely applied to the molecular and biological classification of breast cancers, and several prognostic and predictive signatures have been reported, some of which have been introduced into clinical practice (for example, Onco*type *DX Breast Cancer Assay (Genomic Health, Inc., Redwood City, CA, USA) and MammaPrint assay (Agendia BV, Amsterdam, the Netherlands); for reviews, please see Weigelt *et al. *[21], Sotiriou and Pusztai [22], and Reis-Filho *et al. *[23]). The types of analysis and the data they generate pose a major challenge for the translation of their findings into assays that can be used in routine clinical practice, as the reliability, reproducibility and stability of some have been called into question [23-25]. In terms of preanalytical variables, most of the parameters that affect immunohistochemistry also affect gene expression profiling (for example, time to tissue fixation, time to freezing, length of fixation, type of fixative used and tissue storage). In addition, given that these technologies are based on nucleic acid extracts from tissue homogenates, the variable tumour content may also constitute a confounding factor. Cleator *et al. *[26] demonstrated that varying amounts of non-neoplastic cells in samples subjected to gene expression increases the error rates of multigene predictors, providing direct evidence that the non-tumour content of breast cancer samples has a significant effect on gene expression profiles.

Data analysis of these 'mega-parameter' profiles also poses huge challenges. Microarray technology and bioinformatics/statistics applied to microarray analysis have developed at disparate speeds and this may have led to inappropriate conclusions being drawn and contributed to the first wave of over-optimism and, then, to the subsequent wave of (over)scepticism with this type of technology experienced in the last 10 years [24]. Indeed, when these signatures were first described, there was little awareness of problems of data 'overfitting' and methods for power calculation for microarrays [21,23]. This field has developed rapidly, however, and guidelines regulating how a therapeutically significant gene signature should be developed and validated are now available [27,28]. The chances of success in developing and validating gene signatures will be significantly increased if these guidelines are strictly followed.

## Conclusions

Technical validation and qualification of a biomarker assay may not be as glamorous as biomarker discovery; however, together they comprise the critical lynchpins of translational cancer research. Guidelines for the development and validation of biomarker assays are available. It should be noted, however, that not all aspects of these guidelines may be applicable to the assay to be developed. In these cases, imaginative approaches should be sought while erring on the side of caution. To avoid waste of resources and the use or publication of misleading data, full awareness of the technical challenges in assessment of the robustness of assays and careful evaluation of the context of the assay to be developed are required [29]. Finally, in the reporting of the results, uncertainties should be disclosed and all caveats ought to be voiced. While these issues are undoubtedly challenging and consideration of the numerous potential pitfalls may be discouraging, awareness and avoidance of these problems can allow clinically relevant work of great value to be conducted [29].

## Abbreviations

ER: oestrogen receptor; FFPE: formalin-fixed, paraffin-embedded; HER2: human epidermal growth factor receptor 2; IHC: immunohistochemistry; NEQAS: National External Quality Assessment Service; PR: progesterone receptor; siRNA: short interfering RNA; TMA: tissue microarray.

## Competing interests

The authors declare that they have no competing interests.

## Authors' contributions

CM drafted the manuscript. MD and JSR conceptually designed the manuscript and revised the draft. All authors read and approved the final version of the manuscript.

## Pre-publication history

The pre-publication history for this paper can be accessed here:

http://www.biomedcentral.com/1741-7015/9/41/prepub
